# On AI Approaches for Promoting Maternal and Neonatal Health in Low Resource Settings: A Review

**DOI:** 10.3389/fpubh.2022.880034

**Published:** 2022-09-30

**Authors:** Misaal Khan, Mahapara Khurshid, Mayank Vatsa, Richa Singh, Mona Duggal, Kuldeep Singh

**Affiliations:** ^1^Department of Smart Healthcare, Indian Institute of Technology Jodhpur, Karwar, India; ^2^All India Institute of Medical Sciences Jodhpur, Jodhpur, India; ^3^Department of Computer Science and Engineering, Indian Institute of Technology Jodhpur, Karwar, India; ^4^Post Graduate Institute of Medical Education and Research, Chandigarh, India; ^5^Department of Pediatrics, All India Institute of Medical Sciences Jodhpur, Jodhpur, India

**Keywords:** maternal health, neonatal health, artificial intelligence, lower and middle income countries, machine learning, deep learning

## Abstract

A significant challenge for hospitals and medical practitioners in low- and middle-income nations is the lack of sufficient health care facilities for timely medical diagnosis of chronic and deadly diseases. Particularly, maternal and neonatal morbidity due to various non-communicable and nutrition related diseases is a serious public health issue that leads to several deaths every year. These diseases affecting either mother or child can be hospital-acquired, contracted during pregnancy or delivery, postpartum and even during child growth and development. Many of these conditions are challenging to detect at their early stages, which puts the patient at risk of developing severe conditions over time. Therefore, there is a need for early screening, detection and diagnosis, which could reduce maternal and neonatal mortality. With the advent of Artificial Intelligence (AI), digital technologies have emerged as practical assistive tools in different healthcare sectors but are still in their nascent stages when applied to maternal and neonatal health. This review article presents an in-depth examination of digital solutions proposed for maternal and neonatal healthcare in low resource settings and discusses the open problems as well as future research directions.

## 1. Introduction

Child and maternal health are key components of every country's growth. In the early 1990s, world leaders approved eight Millennium Development Goals (MDGs), including improving maternal health and reducing infant mortality by 2015. Between 1990 and 2015, the programme resulted in a decrease in the number of deaths of women and children; the mortality rate of children under the age of five reduced to half since 1990, and maternal mortality decreased by 45% globally. Even with these advancements, over 830 women and 7,400 babies die every day as a result of difficulties during pregnancy, childbirth, and the postnatal period, totaling an estimated 303,000 maternal and 2.87 million newborn deaths per year. An additional 2.6 million newborns lose their lives to stillbirths. A vast majority of these deaths happened in underdeveloped regions with limited resources, such as Africa and Southeast Asia. In 2015, the World Health Organization (WHO) proposed the Sustainable Development Goals (SDGs), a set of 17 objectives to be accomplished by 2030. The third SGD aims to ensure healthy lifestyles for all people on the planet, including a reduction in maternal mortality to less than 70 deaths per 100,000 live births and neonatal mortality to less than 12 deaths per 1,000 live births (1).

*Maternal health* is concerned with the health of women throughout gestation, childbirth, and the postpartum period. It is not uncommon for women to experience health problems during pregnancy, however these difficulties can impact their health, baby's growth, or both. Women in good health prior to becoming pregnant can also have difficulties. Despite significant advancements in medicine, a high percentage of women still die during and following pregnancy due to a number of factors, including excessive blood loss, infection, high blood pressure, anemia and heart disease. The following are some of the most prevalent complications during gestation and the postpartum period; however, the list is not exhaustive (1).

1. High Blood Pressure: During pregnancy, often is the case of difficulty in the transportation of blood to various parts of the body due to swollen nerves or the arteries becoming too narrow, which causes high pressure in the arteries. This situation is also known as hypertension (HTN), making it difficult for the blood to reach the placenta and provide necessary nutrition to the fetus. It can result in a fetus with stunted growth and put the mother at an increased risk of premature delivery and preeclampsia.
2. Gestational Diabetes: Diabetes affects people of all ages and genders. It is not an infectious disease but surfaces in an insulin deficit individual. Studies have shown that diabetic women are more likely to experience miscarriage, renal failure, cardiovascular diseases, blindness, and other long-term and deadly illnesses (2). For this reason, it is critical to diagnose diabetes in pregnant women as soon as possible.
3. Infections Acquired during Pregnancy: During pregnancy, the immune system of the woman is at its lowest, and she can be exposed to a number of infections. These infections have the risk of spreading to the fetus as well.
4. Preterm Deliveries: Preterm deliveries can lead the infant to be born with many health issues as the final development of the brain along with the immune system takes place in the final term of the pregnancy.
5. Miscarriage or Loss of Fetus: Miscarriage is the condition in which the pregnancy is lost due to natural causes, and they occur very early in the period of pregnancy, having more than 20% of all pregnancies ending in miscarriages.

Ronsmans et al. (3) highlighted that in the most underdeveloped parts of the world, the risk of a woman dying due to pregnancy abnormalities or childbirth, is about one in six, and about one in 30,000 in developed countries. Such a significant gap between developed and underdeveloped countries has led to a failure to reach the goal of MDGs by the end of 2015. The main causes of having a low maternal mortality ratio are clustered around labor, delivery, and the immediate postpartum period, with obstetric hemorrhage, all of which are underestimated in low resource countries.

While maternal mortality has dropped globally, it remains high in low- and middle-income nations such as India, Pakistan, and Nigeria, where maternal health remains a major public health concern. According to the World Health Organization, in 2017, more than 2,95,000 women died both during pregnancy and childbirth. The majority of these maternal deaths can be prevented if a skilled professional is consulted in a timely manner. Reducing preventable deaths should remain a high priority for the global community.

*Child health* can be segregated into two parts: perinatal and neonatal. Perinatal health corresponds to health between the 22nd week of pregnancy (or gestation) and the seventh day following birth. The focus on ensuring good perinatal health is to supplement further neonatal development of the baby in the first month of life after birth. Proper care in these periods is essential to build a healthy foundation for the baby, which corresponds to a healthy childhood and adulthood. The neonatal period refers to the first few weeks of the infant after perinatal. These are the most developing weeks of its lifespan, and without access to proper care by healthcare providers such as neonatologists, pediatricians, family physicians, or nurse practitioners, many complications can arise, hampering the health of the baby. Premature birth, intrapartum problems, and infection are the leading causes of neonatal mortality worldwide. Some of the most significant problem statements related to neonatal health focused on by researchers are as follows:

1. Stillbirths: Stillbirth is a condition in which the fetus dies while it is inside the womb.
2. Intrapartum problems: Premature birth, low birth weight infants, fetal growth restriction, antenatal complications (e.g., anemia, eclampsia) and other factors during delivery (e.g., extended labor, umbilical cord prolapse) contribute to the development of neonatal health risks such as cerebral palsy, learning disabilities, and other abnormalities.
3. Infections: Infections affect people of all ages, but they are particularly risky in infants because their immune systems are still developing, and they are thus, more prone to diseases.

The postpartum period is when the mother and kid adjust to one other. During this time, the mother may experience anxiety, annoyance, and melancholy, which, in most circumstances, can lead to depression. Postpartum Depression (PPD) is a severe health issue which impacts not only the mother but the child and the entire family as well. However, it is common for such a disorder to go undiagnosed (4).

Most infant healthcare devices that support neonatal care are designed for high-resource settings and are either inaccessible or ineffective in low-resource settings. As a result, low-resource environments lack the instruments necessary to support high-quality, holistic infant care. There is an immediate need for newborn medical technologies that are cost-efficient, durable, effective, easy to use and maintain, and can run on a variety of power sources (5). Addressing these challenges requires a deep understanding of the kinds of complications that occur during pregnancy and the reasons for these complications. There is a need for further progress in the field of quick detection and treatment of maternal and newborn health issues in low-resource health centers and settings.

This article examines artificial intelligence-based strategies for developing approaches to improve maternal and neonatal health. Artificial intelligence is an area of computer science that aims to design/develop intelligent machines/models that imitate various aspects of human intellect. These models are capable of performing a variety of tasks, including learning, thinking, and planning, among others. To achieve this, there is a branch of AI called Machine Learning (ML) that consists of a set of tools and techniques for developing such intelligent algorithms. ML includes various methods including supervised (6, 7), unsupervised (8, 9), semi-supervised (10, 11), and reinforcement learning (12, 13).

**Figure 1:**
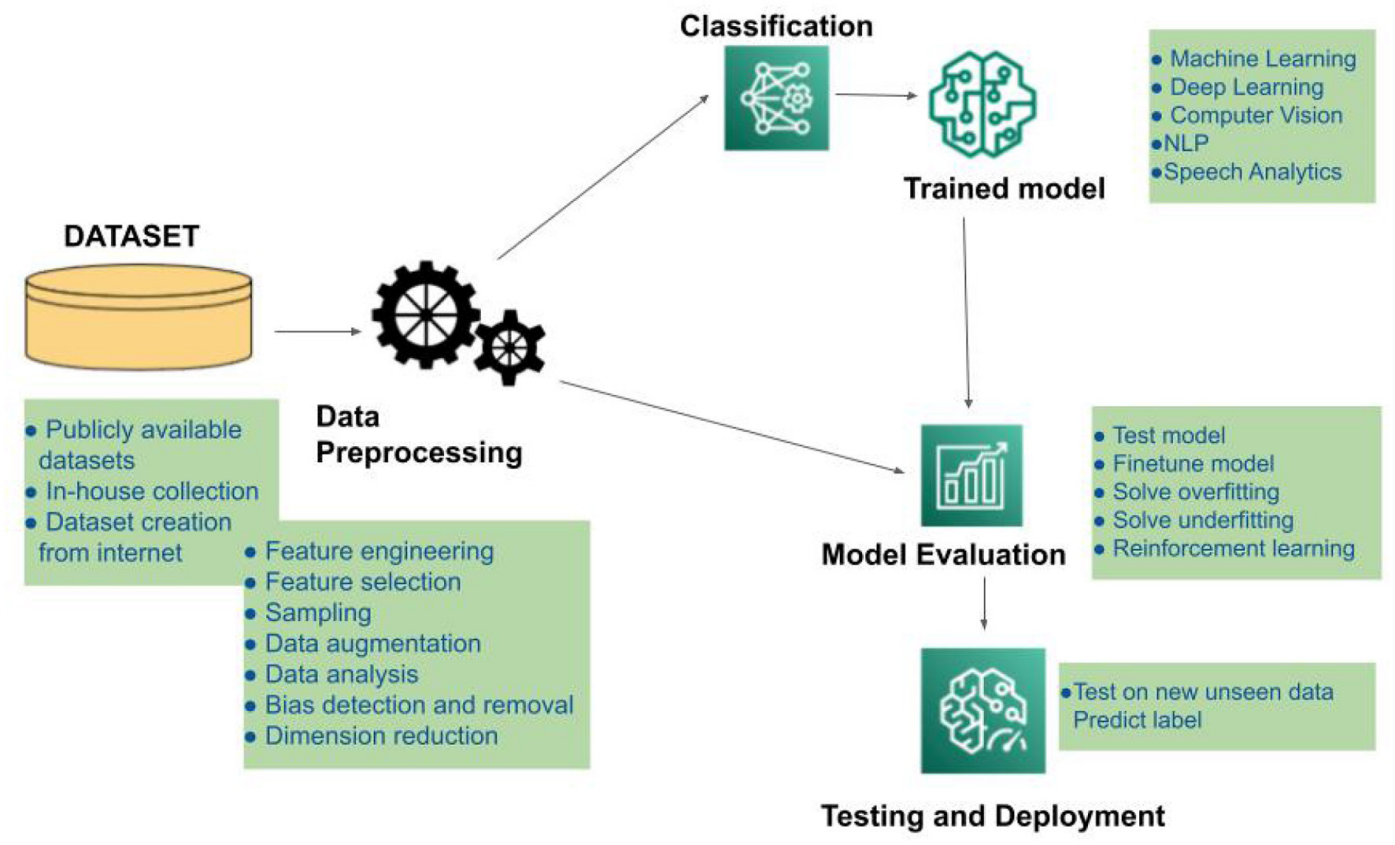
Illustrating different states of an AI based system.

The simplest approach of Machine learning involves using algorithms to evaluate and analyze the collected data and then applying the outcomes of that interpretation to make judgments and predictions about real-world occurrences. Machine learning, unlike conventional software programmes, analyzes large volumes of data to learn how to efficiently perform certain tasks. Figure 1 illustrates the roadmap of an AI-based system in terms of data collection and preprocessing methodologies, model construction, training, evaluation, and real-world testing of the developed framework. Based on their individual learning and assessment methodologies, different types of input data can be processed in a variety of ways to provide the appropriate output. ML methods include supervised, semi-supervised, and unsupervised learning. These algorithms are categorized based on whether or not ground truth labels are available at the time of training. These algorithms can deal with small sample size situations (where the amount of data available is limited) by producing robust and dependable models.

AI based technologies have the potential to evaluate health record data, especially for situations where traditional statistical methods are ineffective. The algorithms are even better for large-scale and high-dimensional datasets. As a result, these algorithms may be utilized to tackle challenges including streamlining care pathways, standardizing medical assessment and diagnosis, discovering patient phenotype correlations, and generating predictive models (14).

### 1.1. Maternal Health Status in Low Resource Settings

Maternal health refers to the well-being of women during various phases, such as during pregnancy (antenatal care), childbirth, and the postpartum period. Taking care of women's health during these periods is crucial in lowering maternal mortality. Direct factors such as significant blood loss, high blood pressure, and obstructed labor or indirect complications such as anemia, depression, and heart disease are the leading causes of maternal death. Maternal mortality refers to the deaths due to pregnancy or while delivering an infant. As per WHO and Elsevier reference module in biomedical sciences, (15), the maternal mortality rate can be measured with the help of the following maternal mortality rate (MMR), which can be defined as the number of maternal deaths in a given time period divided by the number of live births (per 100,000 live births) during the same period:


$$MMR=\frac{number\;of\;maternal\;deaths}{number\;of\;live\;births}\times \frac{1}{100,000}$$

**Figure 2:**
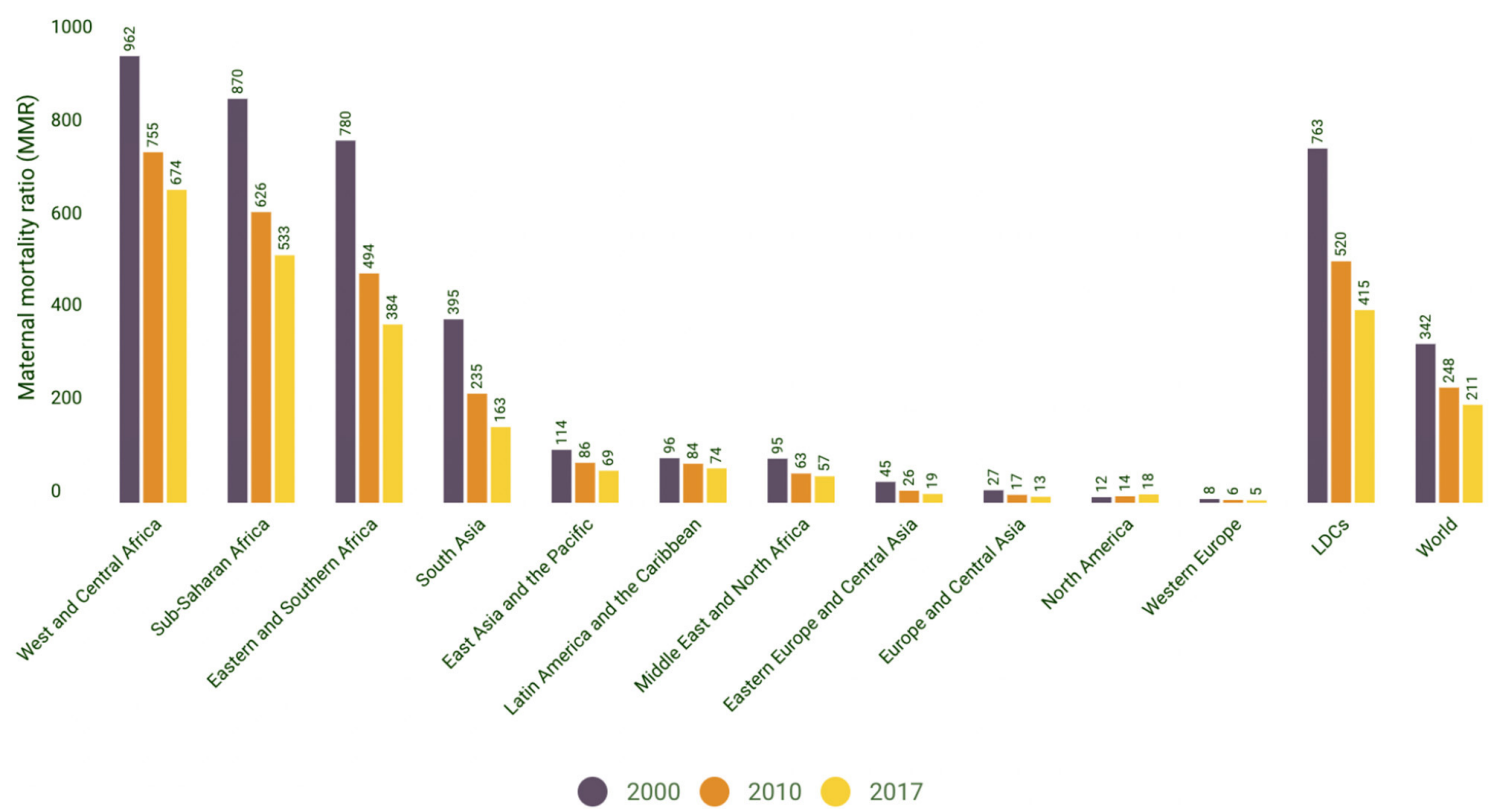
Maternal mortality ratio (MMR) trends by region. Source: World Health Organization, UNICEF, United Nations Population Fund and the World Bank, Trends in Maternal Mortality: 2000–2017 WHO, Geneva, 2019. |UNICEF Data: Monitoring the situation of children and women.

Figure 2 Shows the trends in MMRs across different regions in the world, indicating the highest Maternal Mortality ratios in low-income countries compared to very low MMRs in high-incomecountries.

**Figure 3:**
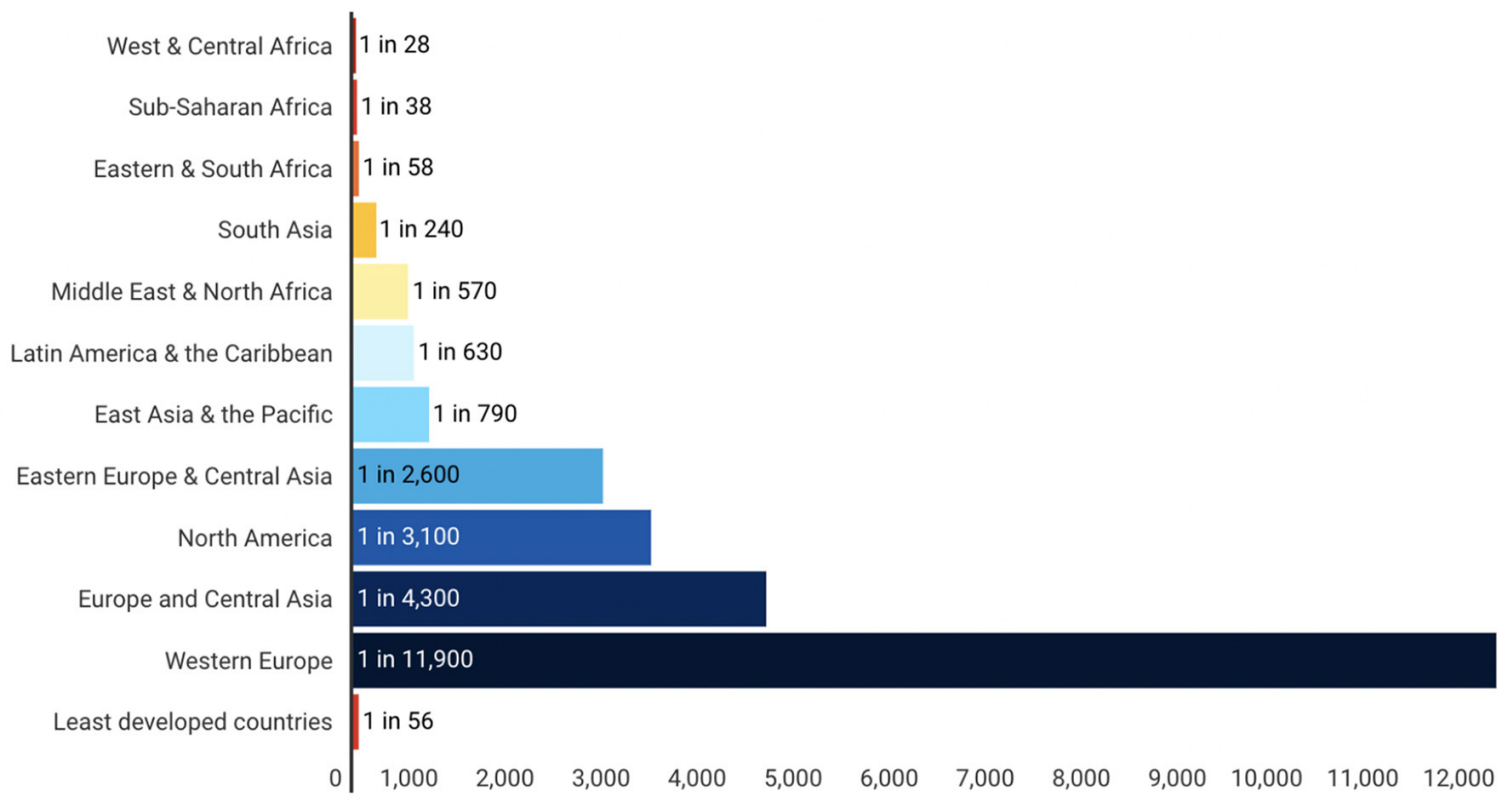
Lifetime risk of maternal death: 1 in X, By region/group. Source: WHO, UNICEF, UNFPA and the World Bank, Trends in Maternal Mortality: 2000 to 2017, WHO, Geneva, 2019. |UNICEF Data: Monitoring the situation of children and women.

**Figure 4:**
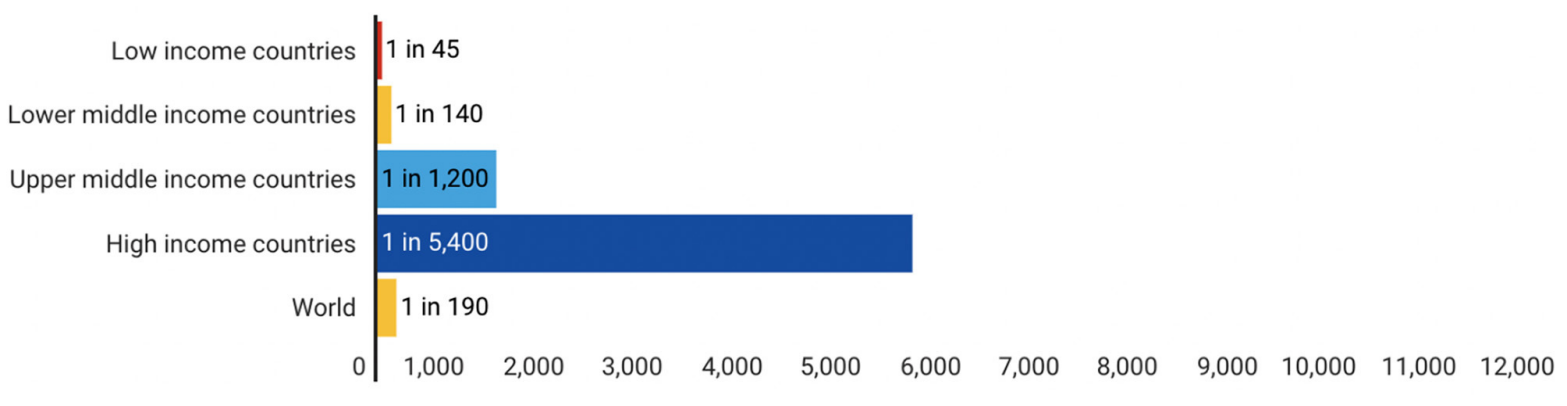
Lifetime risk of maternal death: 1 in X, By income group. Source: WHO, UNICEF, UNFPA and the World Bank, Trends in Maternal Mortality: 2000 to 2017, WHO, Geneva, 2019. |UNICEF Data: Monitoring the situation of children and women.

**Figure 5:**
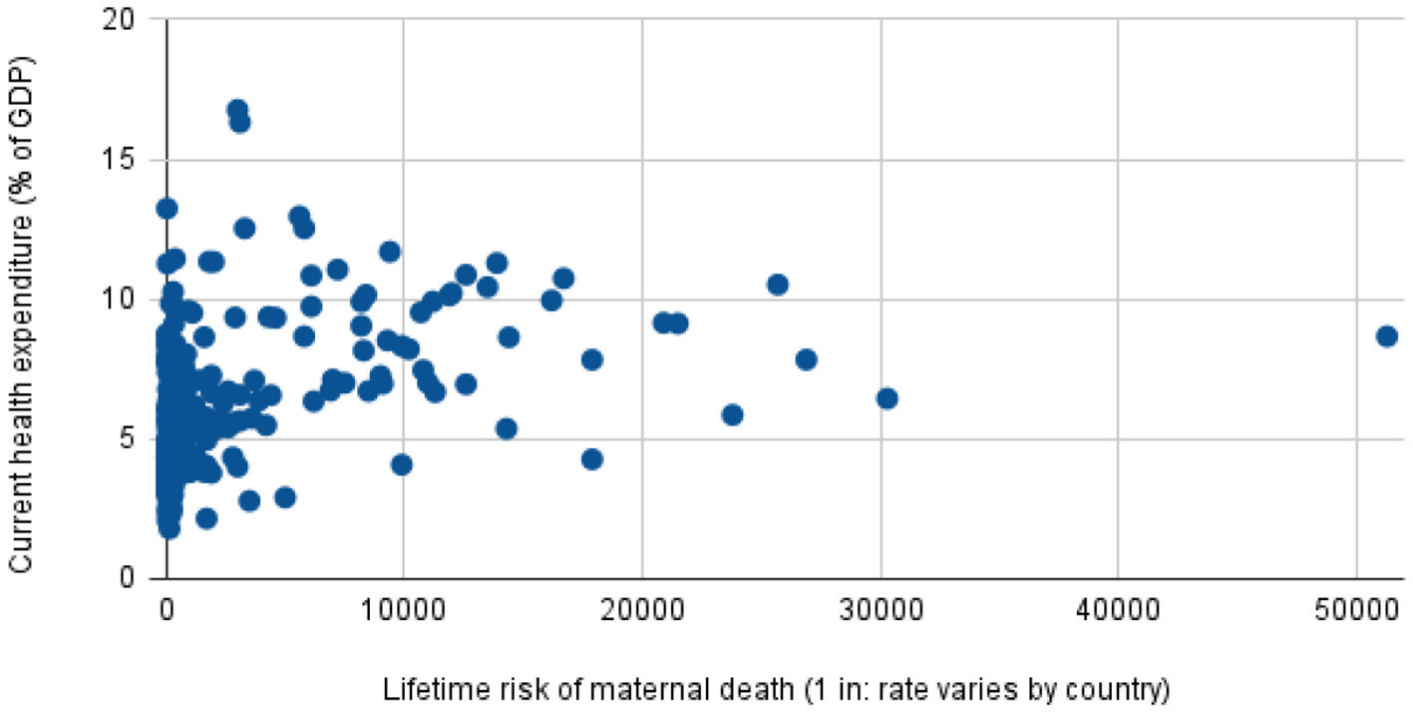
Current health expenditure (% of GDP), by Lifetime risk of maternal death (1 in: rate varies by country) Source: World Bank Data:Current health expenditure (% of GDP), Trends in Maternal Mortality.

Adult lifetime hazard of maternal mortality is defined as the probability that a 15-year-old female will die as a result of a maternal cause over her lifetime. Similar to trends in MMR, the Lifetime risk of maternal death by region/group as shown in Figure 3 indicates that women living in countries with low-resource public health countries face a higher risk of maternal death in their lifetime. This is even substantiated by Figure 4 which further establishes that low and lower-middle-income countries have a significantly high lifetime risk of maternal death. When contrasted with the current health expenditure in health by each country as defined by their GDP, we can although observe in Figure 5 that countries' health expenditure seems not positively or negatively correlated to the lifetime risk of maternal death.

Special testing during pregnancy is required when there is a higher risk of complications. These are usually started between 32 and 34 weeks of pregnancy; however, they can be done sooner if there are many risk factors present such as (i) high-risk pregnancy where the woman has a pre-existing health condition such as cardiac disease or diabetes, (ii) fetal growth problems, (iii) reduced fetal movement, and post-term delivery. The non-stress test, biophysical profile (Fetal heart rate, breathing movements, body movements, amount of amniotic fluid), fetal movement counts, and a Doppler ultrasound check of the umbilical artery are all used to monitor fetal health.

### 1.2. Child Health Status in Low Resource Settings

Neonatal health care is necessary for both categories of childbirths, the mature and the premature. A premature baby is a baby born before 37 weeks of pregnancy. However, high infant mortality rates in low-resource settings are caused by a lack of access to and under-utilization of efficient health systems, which is exacerbated by a plethora of variables such as disparities in coverage, scarce human resources and infrastructure, consultation information, and community/public health systems.

**Figure 6:**
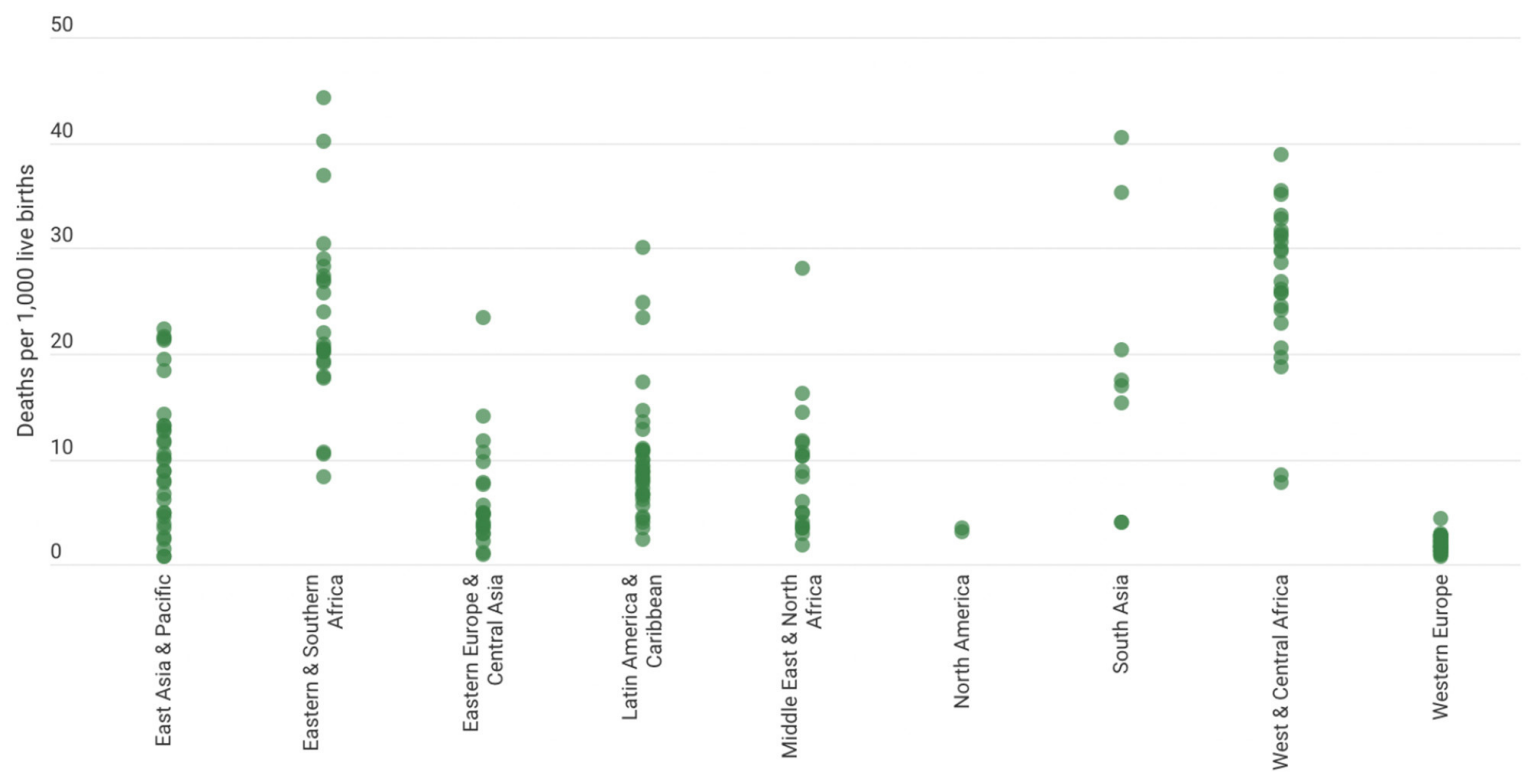
Neonatal mortality rates, by country and region, 2020. Source: United Nations Inter-agency Group for Child Mortality Estimation (UN IGME), 2021|UNICEF Data: Monitoring the situation of children and women.

Neonatal mortality is frequently used as a metric for a core indicator of neonatal health and well-being and is a significant component of overall under-five mortality, as it is defined as death occurring within the first 28 days of life. UNICEF (16) study demonstrates that a poor nation has a higher neonatal death rate than a developed country. Numerous studies have been conducted in this area to determine the factors that contribute to neonatal mortality, including septicaemia, respiratory distress syndrome, premature births, low birth weight, low APGAR (Appearance, Pulse, Grimace, Activity, and Respiration) scores (a quantitative score to measure newborn resilience), low socioeconomic status, cesarean section (C-section) delivery, and neonatal age at admission (17). Figure 6 depicts the disparities in Neonatal Mortality Rates by country and region, highlighting that children born in South Asia and Saharan Africa are most susceptible to illness and mortality in their first month in comparison to any other child born in a high-income, high-resource region/countries. Expanding on the same viewpoint, Table 1 lists the names of the 10 countries with the highest infant mortality rate, with India having the highest number of newborn deaths in the world.

**Table 1:** Top 10 countries with the highest number of neonatal deaths, 2020.

**Country**	**Number of newborn deaths in thousands (90% uncertainty interval)**
India	490 (425–558)
Nigeria	271 (199–374)
Pakistan	244 (198–298)
Ethiopia	97 (77–123)
Democratic Republic of the Congo	96 (56–163)
China	56 (49–64)
Indonesia	56 (45–70)
Bangladesh	51 (45–57)
Afghanistan	43 (32–55)
United republic of tanzania	43 (30–62)

Source: WHO-Fact Sheets, Neonatal Mortality.

These statistics indicate an alarming need for interventions to address this issue to alleviate the current status and promote health and nutrition in children, as years later, these would be the driving generation of the country. To overcome these issues, novel and innovative techniques involving the utilization of relevant digital technology are required.

The rest of the article is structured as follows: Section 2 discusses the role of AI in maternal health, including maternal health monitoring, risks of preterm deliveries and miscarriages, gestational diabetes, complications in females with congenital cardiac diseases, gestational anemia, and postpartum depression. Section 3 discusses the role of AI in neonatal health, including pain assessment, sepsis prediction, neonatal jaundice, and machine learning algorithms for tracking malnutrition. Section 4 discusses the path forward, including economic, societal, and technological barriers, and finally, the summary of the article is given in Section 5.

## 2. Role of AI in Maternal Health

**Figure 7:**
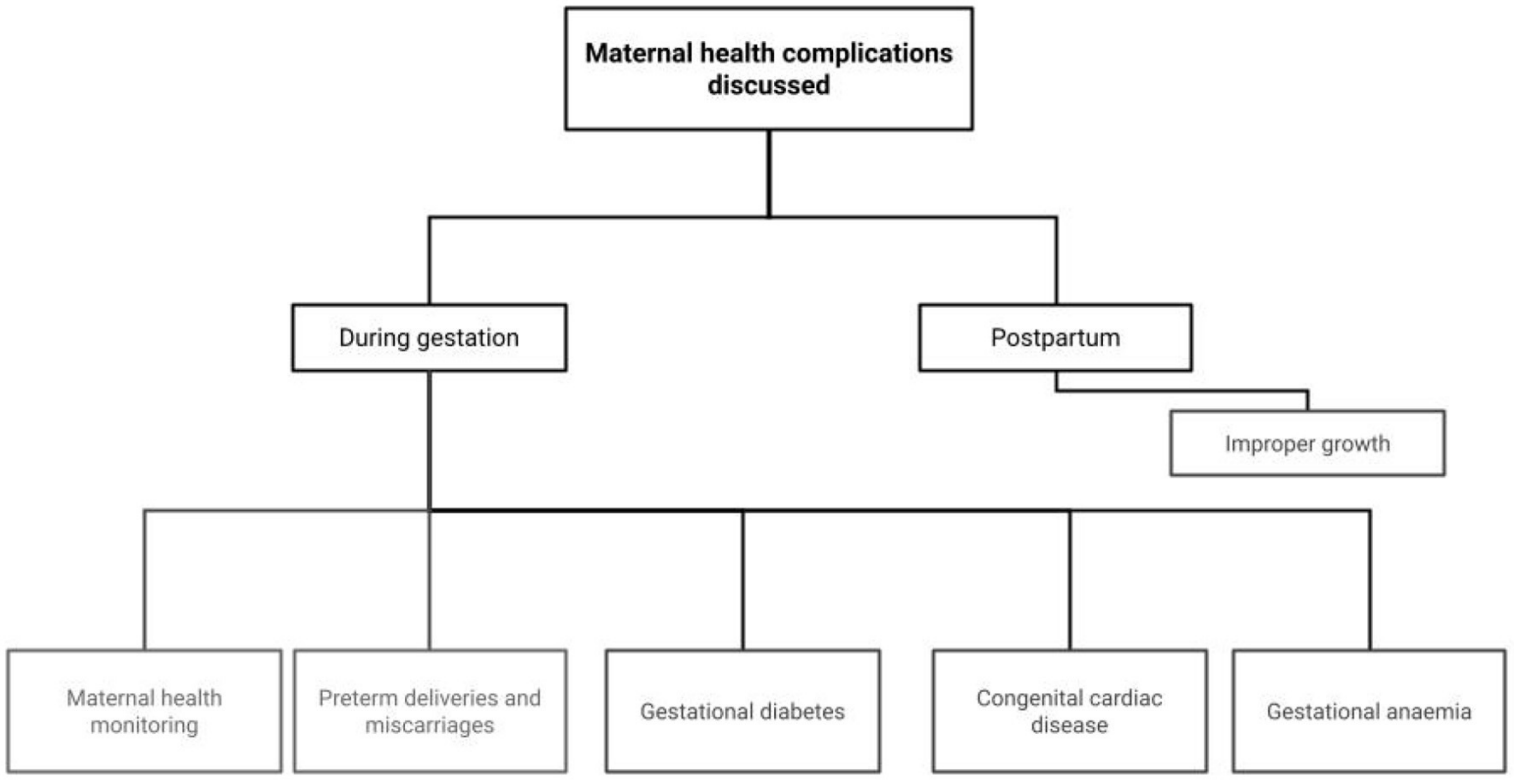
Focus areas on role of AI in complimenting maternal health.

This section reviews the literature on the use of AI to monitor and improve the health of the mother during various phases of pregnancy, childbirth, and postpartum. Timely management of various maternal health issues, including preterm deliveries, miscarriages, gestational diabetes, heart diseases, and postpartum depression, can help in reducing maternal mortality. The main areas of focus for this section are shown in Figure 7.

### 2.1. Maternal Health Monitoring

Maintaining optimum maternal health is vital for the proper growth and development of the fetus. While the mother undergoes several changes during the pregnancy, it is important to monitor maternal health for the signs and symptoms that may indicate a disruption in normal sustenance and functioning of the mother or the fetus. Often during pregnancy, the transportation of blood to all body parts becomes constricted due to swollen nerves or the arteries becoming too narrow, which causes a lot of pressure in the arteries. This situation is also known as hypertension (HTN), which makes it difficult for the blood to reach the placenta and provide necessary nutrition to the fetus. This can cause stunted growth of the fetus and place the mother at greater risk of preterm labor and pre-eclampsia. During pregnancy, the immune system of the woman is also at her lowest, and she is exposed to a number of infections and conditions; and if proper care is not taken, it can even affect the fetus as well. Many of these conditions can be prevented or treated with appropriate pre-pregnancy, prenatal, and postpartum follow-up care (18).

With the widespread adoption of Internet of Things (IoT) technology, building smart IoT devices to support maternal health began to gain traction. Table 2 summarizes some of these approaches. Li et al. (19) presented an IoT platform with wearable technology, cloud computing, and other innovations. They also explored its usage for surveillance and management techniques for gynecology departments in hospitals and homes. The smart maternal platform promises to reduce medical staff workload, raise overall productivity, make things easier for expectant mothers to see doctors, and enhance obstetrical treatment and follow-ups. The results from the questionnaire were analyzed using SPSS statistical software while the use of wearable IoT devices for women during pregnancy is assessed using the chi-square analysis. The p-value compares the experimental and control groups and comes out to be less than 0.05 showing statistical significance. Tracing a similar path, a machine learning approach for predicting foetal wellbeing is suggested through an e-Health application in work done by Akbulut et al. (20) The suggested model was trained using a dataset collected from 96 expectant mothers. Nine binary classification models were trained, validated, and analyzed to forecast overall foetal health. The Random decision Forest (RF) model had the highest accuracy (89.5%), F1-Score (75%), and AUC (Area under the ROC Curve) (95%). In real-world testing, 87% of the consumers performed well. This estimate is adequate to assess foetal health prior to a doctor's appointment.

**Table 2:** Smart devices and applications based maternal health monitoring.

**References**	**Summary**	**Key contribution**	**Dataset used**
Li et al. (19)	Building Smart IoT devices to compliment Maternal health.	A novel IoT framework for smart maternity care leveraging wearable devices and essential technologies along with applications, monitoring and administration modes in-home obstetrics departments. Comprehensive review of the challenges and opportunities in the employment of such frameworks as well as their level of acceptance in the current scenario.	Questionnaire dataset from 315 Chinese participants belonging to 27 provinces. No general obstetrics, gynecology, or other general medical histories relating to prenatal treatment were screened out
Akbulut et al. (20)	The authors suggest an e-Health application with a machine learning algorithm for predicting foetal health.	Pregnant women and physicians can get help from an online assistive system and a prediction system. The impact of specific clinical data parameters of pregnant women on foetal health status was statistically connected with the presence of congenital diseases, and advice for future research were provided.	The suggested model was trained on data from 96 pregnant women. The data came from a maternity questionnaire and three clinical examinations at the RadyoEmar radiodiagnostics facility in Istanbul, Turkey.

### 2.2. Predicting Risks of Preterm Deliveries and Miscarriages

The gestation period is the time span between conception and birth. Throughout this time, the baby develops and grows inside the mother's womb. Gestational age is a word that is widely used during pregnancy to refer to the stage of pregnancy. It is calculated in weeks, beginning with the first day of the woman's last menstrual cycle and ending with the current date. Pregnancy typically lasts 38–42 weeks.

Premature babies are those born prior to the 37th week of pregnancy. Postmature babies are those born after 42 weeks. Preeclampsia is a condition in which a pregnant woman is in danger of preterm delivery and death. Such deliveries are a major issue in underdeveloped countries, mostly due to a lack of timely professional care and awareness of such practices and complications. Therefore, ML/AI-based systems are required, which can be built with training on the obstetrical data (21).

It has been shown in several studies that AI can be used to detect if there is a chance of getting preeclampsia to a very high degree of certainty. However, the current models are only working at high accuracy for early-onset and not post-onset, which has a higher occurrence rate; therefore, there is a requirement for a prediction model with a low false-positive rate and economically feasible predictors that have a higher sensitivity while maintaining the same specificity as others with low-cost predictors (22).

Miscarriage is the condition in which the pregnancy is lost due to natural causes, and they occur very early in the period of pregnancy, having more than 20% of all pregnancies ending in miscarriages. A miscarriage occurs when a fetus dies naturally before the 20th week of pregnancy. The word “stillbirth” refers to the fetus' death after this period. Although there has been a drastic improvement in prenatal care over the years, however, the reality is stillbirths still happen and often go unexplained. With the advent of AI in healthcare, it is possible to identify nearly half of stillbirths antenatally using a combination of existing pregnancy problems, congenital defects, maternal features, and medical history. When compared to logistic regression (LR), ensemble classifiers provided a slight improvement in prediction (23). The importance of addressing the issue of preterm births lies in the impact of such conditions on the family. Both parents may be affected by a miscarriage, and it is impossible to change the result of the pregnancy. Thus, detecting such conditions is very important yet difficult for a novice health worker and requires extra attention from a trained doctor. Miscarriage may only be dealt with by taking particular precautions and preventing it. However, machine learning-based models have made it easier to detect early signs of miscarriages based on time-lapse images of pre-implantation development (24). Preventing premature delivery and detecting preterm labor certainly have significant health and economic implications. Although most efforts have been focused on reducing the impacts of preterm delivery, researchers have also made efforts to predict the risk of preterm birth in pregnant women using machine learning approaches on specific sample signatures. Table 3 summarizes the approaches employing AI in predicting the risk of preterm deliveries.

**Table 3:** Summary of approaches used to predict risk of preterm deliveries.

**References**	**Summary**	**Key contribution**	**Dataset used**
Fergus et al. (25)	Use of Electrohysterography (the analysis of uterine electrical signals) for diagnosing actual labor and predicting premature birth	Unlike previous works in this domain that focus only on detecting true labor using EHG near the days of delivery, this study uses EHG to even predict term and preterm delivery in early pregnancy	Term-Preterm EHG containing 300 records (38 preterm and 262 term)
Hussain et al. (26)	EHG signals are used to detect preterm births with a novel algorithm	The authors describe a unique dynamic self-organized network immune algorithm for categorizing term and preterm records. The article focuses on boosting sensitivity rates, as forecasting preterm delivery is more crucial than misclassifying a term pregnancy	Term-Preterm EHG
Fergus et al. (27)	Proposed a novel self-organized network immune algorithm that classifies term and preterm records	New electromyography features and feature ranking approaches were used to assess their discriminative powers in detecting term and preterm pregnancies. A comparison of seven different neural networks is performed	Term-Preterm EHG
Despotovic et al. (28)	This study investigates the feasibility of predicting preterm birth from EHG recordings made between the 22nd and 25th week of pregnancy	EHG signals based preterm birth prediction using novel features utilising signal's non-stationarity	Term-Preterm EHG
Gao et al. (29)	Deep learning techniques based Extreme preterm delivery(EPD i.e before the 28th week of pregnancy) prediction	Showed that deep learning algorithms could predict extreme preterm birth (EPB) with the help of temporal relationships in electronic health records (EHRs)	Electronic health records
Jehan et al. (30)	Predicting preterm deliveries using the proteomic and metabolomic characteristics	Established a link between omics data and the prediction of preterm deliveries. Provided a method to predict preterm deliveries in early pregnancy (median gestational age of 13.6 weeks as determined by ultrasonography). PTB prediction accuracy was increased by the use of different omics data sets, implying that PTB is a condition that presents in a variety of biological systems	Blood and urine samples collected from 81 pregnant women. The data was examined from December 2018 to July 2019

One such diagnostic/prognostic study conducted by Jehan et al. (30) involved using a machine learning model to predict preterm deliveries using the proteomic and metabolomic characterization of blood and urine samples collected from 81 pregnant women belonging to 5 distinct birth cohorts. The study established a link between omics data and the prediction of preterm deliveries, which is crucial for further research into the said area. The study involved the use of plasma samples analyzed for proteins and untargeted RNA profiling, along with urine samples analyzed for metabolites. The Preterm Birth (PTB) characteristic was described as childbirth before the 37th week of pregnancy. Out of the 81 pregnant women, 39 of them had PTBs (48.1%), and 42 of them had term pregnancies (51.9%). Univariate analysis revealed functional biological differences between the five groups. Each biological data set was subjected to a group-adjusted machine learning method, and the findings were subsequently merged into a final integrated framework. When compared to the models developed for each individual biological modality, the integrated model showed more accuracy and area under the receiver operating characteristic curve (AUROC) of 0.83 (95% CI, 0.72-0.91) than the transcriptomics, metabolomics or proteomics model. The main features of PTB were an inflammatory module and a metabolomic module evaluated in urine that was linked to the metabolism of glutamine, glutamate, and valine, as well as the biosynthesis of valine, leucine, and isoleucine. Preterm birth prediction models have traditionally concentrated on early preterm (28–32 weeks) and intermediate to delayed preterm (32–37 weeks). The bulk of newborn deaths is caused by extreme preterm birth (EPB), which occurs before the 28th week of pregnancy. Gao et al. (29) did a study to address the problem statement and found that EPB can be predicted using deep learning techniques that take into account temporal relationships. It was highlighted that individual predictive models could not outperform ensemble models in performance.

Another such work done by Fergus et al. (25) explores the application of Electrohysterography (EHG) techniques to predict preterm deliveries. The study was based on designing a supervised learning model upon an open-source dataset consisting of 300 EHG records of term and preterm deliveries (31). Using the polynomial classifier, the said approach outperforms previous results, with 96% sensitivity, 90% specificity, and a 95% AUC value with an 8% global error. The results obtained were suggestive of the positive potential of EHG signals in classifying term and preterm pregnancies. More future work in more comprehensively collected datasets was suggested in the conclusion of the research work. Similar studies done on the same Physionet dataset (Term-Preterm EHG) by Fergus et al. (27), Hussain et al. (26) and Despotovic et al. (28) individually explore the application of EHG signals combined with additional features which further improved the performance over shorter time length EHG signals of the suggested models.

### 2.3. Predicting Gestational Diabetes

Diabetes affects people of all ages and genders. It is not an infectious disease but surfaces in an insulin deficit individual. However, it negatively impacts essential organs that it is known as the “mother of all ailments.” Diabetes has more significant implications for women due to their shorter lifespan and poor quality of life. As investigated by World Health Organization (WHO) data, several females with diabetes aren't even aware of being diabetic. Even in high-income countries, gestational diabetes tends to impact about 5–7% of pregnancies (32, 33). In India itself, over 5 million women are affected annually by gestational diabetes, and the rate of such incidences has increased over the decade (34). Gestational diabetes also tends to show increased prevalence across specific ethnicities and racial subgroups (35, 36). This condition can be inherited, especially if the mother is diabetic at the time of being pregnant. Diabetic women are more likely to experience miscarriage, renal failure, cardiovascular diseases, blindness, and other long-term and deadly illnesses (2). For this reason, it is critical to diagnose diabetes in pregnant women as soon as possible.

Gestational diabetes affects pregnant women when the pancreas are unable to produce enough insulin. For a decade, it has been one of the top challenges for ML researchers to identify and diagnose diabetes. For the same purpose, many different algorithms have been employed to date to serve the application (37), ranging from classical machine learning (38–41) to deep learning methods (42, 43). Many researchers also came up with custom methods for diabetes prediction (44, 45).

It was thus established that AI could be used in order to detect Gestational diabetes in pregnant women as early as the first trimester. With the help of variables such as age, family history of diabetes in a first-degree relative, multiple pregnancies, previous gestational diabetes history, fasting plasma glucose, HbA1c, triglycerides, and other laboratory indexes during the first trimester can be used to build a neural network-based model for detecting early signs of gestational diabetes. Table 4 summarizes some of the AI-based approaches for predicting gestational diabetes. For diabetes prediction in pregnant women, Debata and Mohapatra (46) conducted a study. They designed a machine learning model utilizing Chaotic-Jaya (CJaya) algorithm and Extreme Learning Machine (ELM) and trained it on the Pima Indian diabetes dataset. The hybrid approach was named as CJaya-ELM model. The proposed CJaya-ELM model achieved the greatest accuracy of 96.87%, sensitivity of 1, area under the curve (AUC) value of 0.9782 and specificity of 0.9688. The results indicate that the CJaya-ELM model successfully classifies both positive and negative samples from the Pima dataset and outperforms other models such as basic and other modifications ELM, Multi-Layer Perceptron (MLP), CJaya algorithm and Teaching Learning Based Optimization algorithm (TLBO). Another study conducted by Araya et al. (47) used principal component analysis (PCA) on a dataset obtained from 39 pregnant mothers in Concepcion(Chile); the authors found a link between specific thyroidal hormone signatures and Gestational Diabetes. Despite the exploratory nature of these findings and the limited sample size, the correlation is strong enough to predict future behavior. To improve gestational diabetes diagnosis, a multivariate analysis on a larger dataset can be used. Diagnosis of pregnant females with Gestational Diabetes Mellitus (GDM) who need insulin therapy may change their treatment to include more regular monitoring and perhaps preventive services. The goal of a prospective cohort analysis done by Eleftheriades et al. (48) was to create a predictive machine learning-based model for insulin therapy in GDM women. The Classification and Regression Trees (CART) machine learning technique was used to evaluate data from 775 female patients with GDM according to the IADPSG criteria. This basic model demonstrated that we could accurately anticipate the requirement for insulin treatment based on maternal factors such as BMI and the results of an Oral Glucose Tolerance Test (OGTT). Women who are overweight and have an abnormal OGTT initial blood glucose level are more likely to develop gestational diabetes. The prediction model's AUC score for internal and external validation was 0.74 and 0.77, respectively. Another population-based prospective cohort study on a similar subject conducted by Liu et al. (49) intended to construct a prediction model using a dataset collected from 19,331 Chinese women who are pregnant. The risk indicators obtained during registration as pre-pregnancy BMI, maternal age, fasting plasma glucose at the time of registration, and alanine aminotransferase concentration were evaluated and used to build the machine learning model based on the eXtreme Gradient Boosting (XGBoost) approach. Compared with conventional methods like logistic regression, The XGBoost model outperformed the approach in terms of performance with a higher AUR score (0.742 vs. 0.663, *p* < 0.001).

**Table 4:** Summary of approaches used to predict risk of gestational diabetes.

**References**	**Summary**	**Key Contribution**	**Dataset used**
Debata and Mohapatra (46)	Diabetes diagnosis in pregnant women utilizing a hybridized chaotic-jaya extreme learning machine model	Model achieved a sensitivity of 1 and specificity of 0.9688 which helps to classify both positive and negative classes with exceptional accuracy	Pima Indian diabetes dataset All cases here are females above the age of 21 who are of Pima Indian ancestry. One target variable, Outcome, is included in the datasets. The patient's BMI, insulin level, age, and previous pregnancies are all predictor variables.
Araya et al. (47)	Using machine learning; this study sought to see if there was a link between the maternal thyroid profile and gestational diabetes throughout the first and second trimesters	Found correlation between thyroidal patterns and Gestational Diabetes	Anthropometric and clinical variables of Thirty-nine pregnant women from Concepcion (Chile). The study has analyzed data of subjects from 12 to 28 weeks of pregnancy
Eleftheriades et al. (48)	Prospective cohort analysis to create a predictive machine learning-based model for insulin therapy in GDM women	Demonstrated that we could accurately anticipate the requirement for insulin treatment based on maternal factors such as BMI and the results of an Oral Glucose Tolerance Test (OGTT). Showed insulin therapy is required by 15-30% of women with Gestational Diabetes Mellitus (GDM). Women who are overweight and have a fasting blood glucose of 98 mg/dl or higher need to be closely monitored and exercise more	775 female patients with GDM according to the IADPSG criteria
Liu et al. (49)	Population-based prospective cohort study to construct a gestational diabetes prediction model	Demonstrated that lifestyle adjustments can significantly reduce the risk of gestational diabetes mellitus prior to the 15th week of pregnancy. The XGBoost approach does not necessitate meticulous data cleaning or preparation, such as exception scaling and collinearity	19,331 pregnant Chinese women with gestational age less than 15 weeks

### 2.4. Predicting Development of Complications in Females Suffering With Congenital Cardiac Disease

Females with congenital cardiac disease are characterized to be at a higher risk of experiencing adverse medical conditions during pregnancy. As a result, Chu et al. (50) conducted a retrospective analysis to develop two machine learning-based prediction models for mothers and their children, which could help physicians adapt special care and therapy for expecting women suffering from congenital cardiac diseases. The summary, key contribution and the dataset details of this approach are given in Table 5. Such models are particularly well-suited for clinical usage in developing nations, where there is a lack of prenatal counseling and pregnancy monitoring infrastructures. The study included 213 patients falling within the criteria of study who delivered birth after 7 months of pregnancy at Shandong University's Qilu Hospital in China. Univariate and multivariate logistic regression analysis was employed for developing risk prediction algorithms for women and infants. The authors also created two nomogram lists for each patient to forecast the specific risk of complications. The developed models showed high accuracy (76–86% in maternal model and 75% to 80% in neonatal model), implying that they are clinically employable and highlight a substantial correlation between high factors and unfavorable maternal and newborn outcomes.

**Table 5:** Summary of approaches used to predict risk of complications in women with congenital cardiac disease.

**References**	**Summary**	**Key contribution**	**Dataset used**
Chu et al. (50)	Two Machine learning-based prenatal risk prediction models were developed for both unfavourable maternal and newborn outcomes, which could help clinicians adapt precise care and treatment in pregnant women with congenital heart defects	Well suited model for prenatal counseling and pregnancy monitoring in low resource settings. The Maternal model has seven high-risk factors: NYHA class, Eisenmenger syndrome, pulmonary hypertension, left ventricular ejection fraction, sinus tachycardia, arterial blood oxygen saturation, and gestation duration. Eisenmenger syndrome, preeclampsia, and arterial blood oxygen saturation were revealed as high-risk indicators in the newborn model	213 patients at Shandong University's Qilu Hospital who gave birth after 28 weeks of pregnancy

### 2.5. Predicting Gestational Anemia

Anemia is related to impaired cognitive and motor development in children and adults, hence affecting the economic growth of countries. Anemia during pregnancy is also connected with unfavorable reproductive outcomes, including preterm birth, low birth weight infants, and diminished iron storage for the newborn, which may result in impaired development. Failure to address anemia may impact the health and quality of life of millions of women, as well as the development and learning of children, and thus, it is important to develop more technologies for timely diagnosis and monitoring of gestational anemia. Table 6 summarizes some of the approaches that help in predicting gestational anemia.

**Table 6:** Summary of approaches used to predict gestational anemia.

**References**	**Summary**	**Key Contribution**	**Dataset used**
Anggraeni and Fatoni (51)	Early detection of anemia during gestation	Development of a Non-invasive self-diagnostic technique. Use of smartphone camera-based prediction suitable for low-resource settings. More objective detection compared to contemporary visual assessment of anemia	Blood samples and palpebral image of 20 pregnant women between the age of 20–36 years with blood types A, B, AB, and O

A study based in Indonesia by Anggraeni and Fatoni (51) explores early detection of anemia during gestation in order to reduce the cases of postpartum hemorrhage. The study aims to build a non-invasive self-care anemia diagnosis system employing a smartphone camera for palpebral color monitoring. The color intensity RGB signals were then quantified with the Colorgrab software (Loomatix) and correlated with the hemoglobin concentration of the specimens, whose standard hemoglobin concentration was determined using the conventional Spectrophotometer method. A high correlation of red color intensity was shown with the help of linear regression. This exploratory investigation could be seen as an early detection method for anemia, as it is claimed to be more objective than the conventional ocular examination.

Ren et al. (52) further highlighted the fact that machine learning methods outperform standard logistic regression models (2018), which extended the use of machine learning to birth outcomes and air quality studies. In two ML-based models, a pregnant mother's exposure to PM10 was recognized as the most potential risk factor for Congenital heart defects. Their models consistently showed that exposure to fine particulate matter raises the chance of congenital cardiac abnormalities in children.

### 2.6. Predicting Postpartum Depression (PPD) and Anxiety

Various complications of pregnancy also cause women to have anxiety and depression attacks, resulting in a stressful scenario for both the mother and the newborn. Due to a shortage of licensed health practitioners, the mental health system faces a clear capacity restriction. Only three people with mental health concerns have secure access to the system for every ten people (53).

As per the current trends, pregnant women can easily be diagnosed with depression with the help of AI models based on just the voice of the women. According to the study conducted in Borders (54), 87% to 94% of US women report at least one health problem immediate postpartum period, including depression and anxiety, and in the second category (anxiety), the usual response of women is encountered with stress. According to Fisher et al. (55), PPD affects 10-15% of women worldwide, with the number rising to 18% - 25% in low- and middle-income nations. The greater rate is due to the population's cultural and traditional traits (56). There are methods for PPD screening that are accessible; however, they are largely intended for patients who are already having depressive symptoms. Research should focus on developing methods for predicting the risk of developing PPD in people who don't show any signs of depression. To keep moving in this direction, researchers take into consideration a variety of characteristics as well as clinical factors when predicting the risk of developing PPD. Table 7 summarizes the approaches that employ AI while proposing approaches to predict PPD.

**Table 7:** Summary of approaches used to predict postpartum depression using machine learning.

**References**	**Summary**	**Key Contribution**	**Dataset used**
Tortajada et al. (57)	An approach to predict PPD using MLP where the authors have used geometric mean while calculating accuracy	To predict the PPD during the first 32 weeks following childbirthUsed pruning methods to identify the influence of each of the variable on the model performance	Collected data of 1,397 women from 7 Spanish hospitals
Sword et al. (58)	Studied the relationship between mode of delivery and PPD	This study concluded that there is no association between mode of delivery and PPD In addition to common PPD indicators, this work identified more indicators such as unmet learning needs, maternal readmission to hospital, and urinary incontinence.	Collected data of 2,560 women having age >= 16 years from 11 hospitals in Ontario, Canada
Jimenez et al. (59)	An approach to detect the risk of PPD during the first week postpartum by employing socioeconomic, psychiatric, and easy-to-answer questionnaires as variables	This work presents a questionnaire-based clinical decision system to classify the women suffering from PPD This app can be used by both clinicians and the females who had just given birth	Collected data of 1,397 women from 7 Spanish hospitals during an 11-month period
Natarajan et al. (60)	used functional gradient boosting methods to predict PPD using non-clinical data	Identified the features that help in early prediction of PPD ML algorithms have the potential to predict the women suffering from or are at the risk of developing PPD	Facebook groups and Twitter
Fatima et al. (61)	Proposed a generalized approach for the PPD using data from social media text	Studied the relationship of posts (textual features) with the PPD and with general depression This study has limited applicability as the dataset is not complete in terms of not being sure about the participants who took part are actually suffering from PPD	Posts from Reddit
Shin et al. (62)	Studied the effects of nine ML algorithms to predict the PPD	Evaluated various machine learning algorithms and found that RF achieves highest accuracy for the task of predicting PPD Handled the data imbalance problem that makes the models robust	Data from PRAMS (Pregnancy Risk Assessment Monitoring System)
Betts et al. (63)	Proposed an approach to identify the women at risk of postpartum psychiatric admission	Explored how big data can be used with ML algorithms for this task. This can help the clinicians to predict the women at risk of developing PPD	Administrative health data
Zhang et al. (64)	Proposed an approach to detect PPD during pregnancy	Using routinely gathered EHR data, this approach can assist doctors in identifying women who are at risk of developing PPD. This model identifies comorbid indicators such as palpitations, hypertensive disorders vomiting during pregnancy, diarrhea and hypothyroidism which can be associated with PPD	Two electronic health records each containing data of 15,197 and 53,972 women, respectively
Andersson et al. (65)	Evaluated a range of ML methods to predict PPD	Extremely randomized trees were able to achieve a well-balanced specificity (75%) and sensitivity (72%), making the prediction model more robust to be used in addition to clinical method Studied the subgroups with previous depression history (before or during pregnancy) in predicting the PPD	Data is obtained from "Biology, Affect, Stress, Imaging and Cognition (BASIC) cohort study conducted at Uppsala University Hospital, Sweden.

Tortajada et al. (57) devised a method for predicting PPD based on MLP. The authors employed four models and calculated the geometric mean of accuracy to assess model performance. The authors came to the conclusion that the models may predict PPD in the first 32 weeks after childbirth. The model was able to achieve an accuracy of 81%. Fatima et al. (61) conducted a similar study to predict PPD using social media text. The authors showed that Multi-layer Perceptron (MLP) outperformed Support Vector Machine (SVM) and Logistic Regression (LR) in prediction when using a hold-out validation technique by achieving an accuracy of 81%.

Jimenez et al. (59) have presented a method for predicting PPD during the first week following childbirth. In this study, the authors employed socioeconomic, psychiatric, and easy-to-answer questionnaires as variables. A number of classifiers were used for classification, including Naive Bayes (NB), Linear Regression (LR), Support Vector Machine (SVM) and Artificial Neural Network (ANN), with NB outperforming the others. Furthermore, the authors have created an app that includes a questionnaire that can be completed by patients or physicians who want to keep track of their patients. The approach gives an adequate level of sensitivity and specificity (both close to 0.73), and is also simple to interpret, according to the findings of the experiments.

Zhang et al. (64) have studied the risk of developing PPD among pregnant women. Various features, including the patient's demographic, mental health history, obstetric complications and many more, are used in this work. The authors used a sequential forward selection strategy to find the best set of traits. Grid search was used to identify hyperparameters for the predictors, including Random Forests (RF), Decision Trees (DT), XGBoost, regularized LR, and MLP. The proposed approach achieved an AUC of 0.937 (95% CI 0.912–0.962) and 0.886 (95% CI 0.879–0.893) in the development and validation sets. Experimental analysis demonstrates that this approach can reduce the burden of identifying the risk of PPD.

Natarajan et al. (60) have proposed using functional gradient boosting methods to predict PPD using non-clinical data. The authors have used various classifiers and calculated various performance metrics such as ROC, precision, recall and also metrics to handle class imbalance problems for them. It is reported that the gradient boosting method outperforms the other classifiers and achieved an ROC of 0.952 with a precision of 0.920. Further analysis of the experiments demonstrates that the ML algorithms can accurately predict PPD. Shin et al. (62) have proposed a method for predicting PPD. The authors used nine algorithms, with the best results coming from RF, Adaboost, GBM, and SVM. To resolve the data imbalance and avoid overfitting, the authors used Synthetic Minority Oversampling Technique (SMOTE) and cross-validation. After extensive experimentation with various classifiers, it is found that random forest outperforms the other classifiers and achieved an AUC of 0.884. The authors also concluded that life stress and a history of depression are the two most important factors in predicting PPD. Betts et al. (63) used a gradient-boosting approach that outperformed the LR and elastic net methods. The approach is likely to learn the complex and non-linear relationship within the data, since the experimental results produced an AUC of 0.80 (95% CI = 0.76–0.83).

Andersson et al. (65) looked at a variety of machine learning algorithms for predicting the probability of acquiring PPD. To evaluate the model's performance, the authors used clinical, demographic, and psychometric data. The extremely randomized trees method provides the highest accuracy of 73% and well-balanced sensitivity and specificity of 72 and 75%, respectively. Furthermore, the scientists concluded that depression and mental health difficulties had a major impact on PPD. To verify the relationship between PPD and the mode of delivery, Sword et al. (58) studied the relationship between the risk of getting PPD and the method of delivery. Social support, maternal age, previous pregnancy, and many other factors were considered by the authors. The authors have performed screening after 6 weeks following the hospital discharge. According to the results, there is no link between the mode of delivery and the PPD.

Although the research community is focusing on developing automated approaches to promote AI in maternal health, some limitations still need to be addressed. The proposed approaches should be bias-free (i.e., they should not represent any particular section of the society), ethnicity-agnostic, and explainable. In order to build trust in the AI systems, the end-users should be well aware of the working of the algorithm while making any prediction.

## 3. Role of AI in Neonatal Health

**Figure 8:**
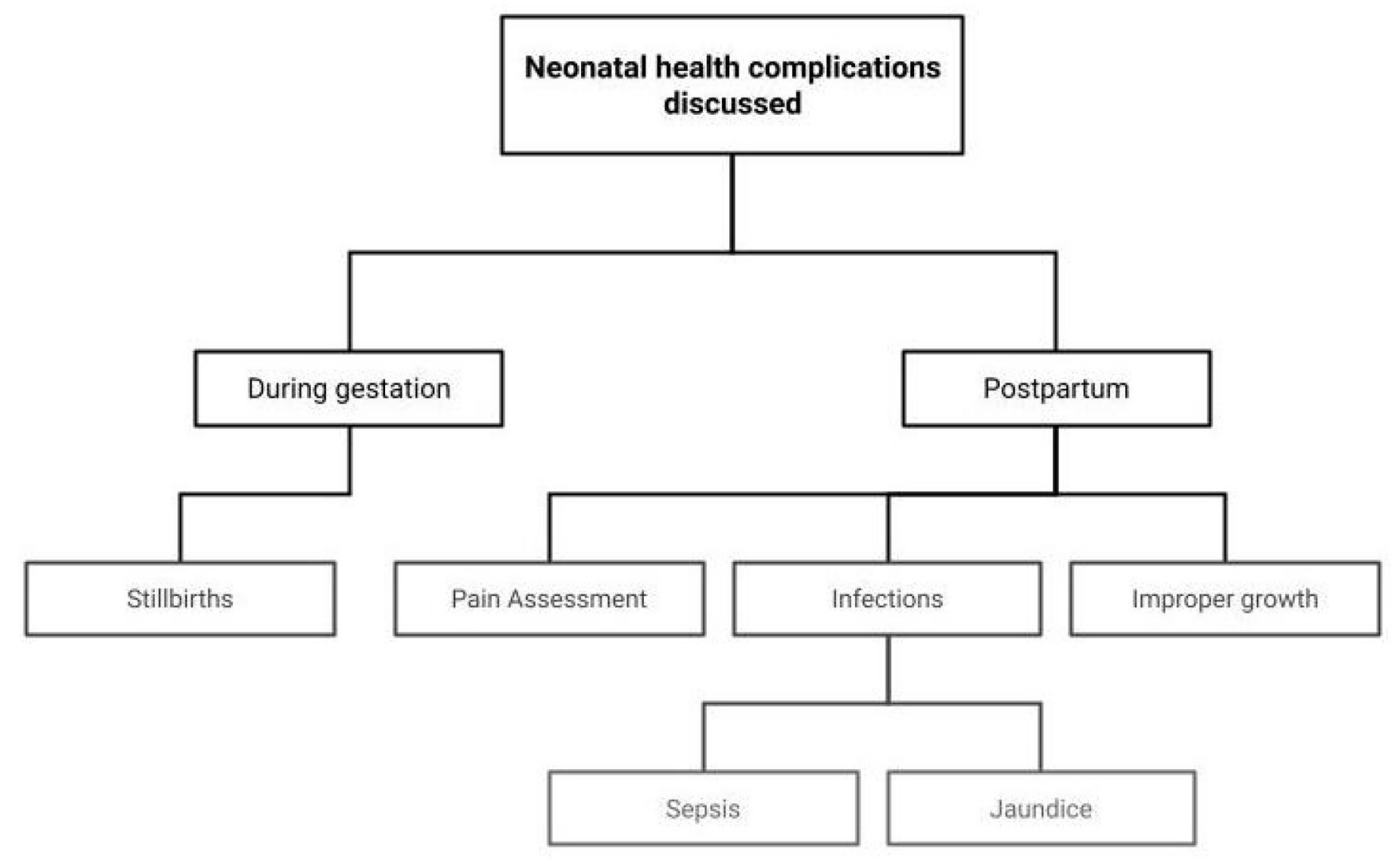
Focus areas on role of AI in supporting Neonatal Health.

This section examines the research on the use of AI to improve newborn health. Following birth, the baby may develop a variety of health problems that necessitates prompt evaluation and treatment. It aids in lowering the rate of child mortality as well as reducing the severity of the implications if left untreated. Pain assessment, sepsis prediction, jaundice, and malnutrition tracking are just a few of the many health issues that require a timely diagnosis. The subsections that follow go into the role of AI in treating these disorders. The key focus areas of this section are represented in Figure 8.

### 3.1. Pain Assessment in Neonates

Pain is a defensive mechanism that is activated in response to any physical or potential tissue damage (66). The pain assessment can assist the caregiver in getting a greater understanding of the patient's medical state and making an accurate diagnosis. However, this process is difficult for infants because of their lack of communication. Ineffective pain management can result in persistent neuroanatomical and developmental abnormalities, as well as learning difficulties (67, 68). As a result, automated pain assessment systems based on behavioral and other physiological factors are required. Researchers are working on techniques that make use of these features, as seen in Table 8.

**Table 8:** Summary of approaches used to predict neonatal pain using AI.

**References**	**Summary**	**Key Contribution**	**Dataset used**
Zamzami et al. (69)	This work devises an approach to predict neonatal pain using facial strain by using various machine learning classifiers including SVM and KNN	This system can be helpful both in hospitals and homes by allowing continuous monitoring of the neonate.	Collected data of 10 infants older than 30 gestational weeks during acute and chronic pain.
Zamzmi et al. (70)	A multi-modal neonatal pain assessment system utilizing behavioral and physiological pain indicators is proposed in this work	The authors utilized multiple pain indicators such as facial expression, body movements and the vital signs to design a multimodal system to assess pain in neonates Experiments reveal that combining multiple pain indicators makes the system more robust and accurate	Collected data of 18 infants (having an average gestational age of 36 weeks) during the routine painful procedure at Tampa General Hospital
Zamzmi et al. (71)	An automated multi-modal system is proposed by including facial expressions, body motion and vital signs	Developed a multimodal system including crying sounds in addition to facial expression, body movement and vital signs for assessing neonatal pain. Can act as a non-invasive and fast method of neonatal pain assessment	Collected data of 18 infants (having average gestational age of 36 weeks) during the acute episodic painful procedure
Zamzmi et al. (72)	To propose a cost-effective pain assessment system using smart sensors and ubiquitous computing to resource-restricted areas	This work uses transfer learning for the automatic assessment of pain. Can be helpful for caregivers both at hospitals and in homes	Collected data during painful procedures of 31 neonates having an average gestational age of 36 weeks at Tampa General Hospital
Zhi et al. (73)	The authors proposed a neonatal pain assessment system by utilizing dynamic facial texture and geometric features from video sequences	This work presented an approach for neonatal pain assessment by combining the collected video sequences' geometrical and temporal facial features. The results demonstrate that this method can be helpful in NICU's to monitor the infants for pain continuously.	Collected data from 31 infants during painful procedures such as heel lancing for 5s at NICU at Tampa General Hospital. The average gestational age of the infant was 36.4 weeks
Zamzmi et al. (74)	Evaluated a deep network, N-CNN for neonatal pain assessment	A light-weight CNN is evaluated that helps in the automated assessment of neonatal pain The findings of using N-CNN are promising, demonstrating that it may be used to supplement to the current standard of pain assessment.	Collected data (video, audio and other vital sign readings) of 31 infants both in resting position and during painful procedures at Tampa General Hospital and USF.
Salekin et al. (75)	A multi-channel network is proposed in this work that uses facial expressions and body movements, also incorporated temporal information using LSTM	A system that uses facial expressions and body movements, the visible indicators, can help caregivers assess neonatal pain. There is a strong correlation between assessing pain using face and body features	Collected data of 31 neonates with an average gestational age of 35.9 weeks during heel lancing and immunization
Zamzmi et al. (76)	Proposed a neonatal pain assessment using physiological and behavioral features with various fusion schemes. Also, proposed a neonatal pain dataset, NPAD	This work generates pain scores by fusing multiple pain indicators, and the results demonstrate the feasibility of using this approach which can be helpful to assess pain Introduced a neonatal pain assessment dataset	Collected dataset of 40 neonates during procedural pain and post-operative pain with a mean gestational age of 35.9 weeks
Salekin et al. (77)	Proposed a crying sound based neonatal pain assessment system where the sounds are converted to spectrogram images	Evaluated the N-CNN to assess neonatal pain using crying sounds as a modality The proposed approach analyzed sounds at baseline and during painful procedures and gave promising results, hence acting as an alternative to the current assessment method.	Collected data (video, audio and other vital sign readings) of 31 infants having an average gestational age of 35.9 weeks
Salekin et al. (78)	The authors proposed an approach for assessing post-operative pain in neonates by using bilinear CNN and LSTM	Studied the use of deep learning in estimating the post-operative pain Used LSTM to continuously monitor the temporal changes in neonates for estimating pain intensity	Collected data (visual, vocal and physiological) of 45 neonates at Tampa General Hospital, COPE acute dataset, and post-operative dataset
Ashwini et al. (79)	Proposed an approach by using deep features with SVM for neonatal pain assessment	Studied the use of deep features with a machine learning classifier in designing a model for neonatal cry classification. SVM with RBF kernel gives the best performance for this task.	Collected data of infants aged between 1 and 10 days (from NTU Hospital, Taiwan)
Salekin et al. (80)	A multi-modal approach for neonatal post-operative pain assessment by using spatio-temporal approach is being proposed	Compared the performance of both unimodal and multimodal for this task. The performance gets improved using temporal information	Used USF-MNPAD-I (University of South Florida Multimodal Neonatal Pain Assessment Dataset) consisting of 45 neonates having gestational age ranging from 30 to 41 weeks

Zamzami et al. (69) have proposed an approach that uses facial strain to predict neonatal pain. The authors gathered in-house data by recording 10 newborns during painful procedures such as heel lancing. Face detection in newborns is difficult due to occlusion by a hand or pacifier, as well as unpredictable movements; the authors manually identified facial landmarks. The authors used k-nearest neighbors (KNN) and SVM to train for classification and attained an accuracy of 96 and 94%, respectively.

Zamzmi et al. (70) have proposed a multimodal pain assessment approach for neonates that includes both physiological and behavioral pain indicators. The authors also evaluated the unimodal approach by focusing just on facial images. The strain magnitude was measured using optical flow estimation, and the strain was calculated using flow vectors. The results demonstrate that the unimodal system employing facial expression achieved the highest accuracy of 88%. However, while combining various pain indicators, the model achieved an overall accuracy of 95%. To add more features, the authors integrated bodily movements and vital signs such as breathing rate, heart rate, and oxygen saturation level. The authors concluded that facial expression attained the highest accuracy in a unimodal pain assessment method. For the multimodal approach, the authors used majority voting and chose the class with the highest confidence score as the final pain assessment. These results suggest the efficacy of using multiple pain indicators while developing the neonatal pain assessment system.

Zamzmi et al. (72) developed a smart and accessible system employing AI and ubiquitous computing to boost healthcare in rural areas and provide a cost-effective approach for neonatal pain assessment. Using smart sensors, the proposed approach can continually monitor the newborns and report to the caregiver. The authors employed the VGG-Face feature extractor and the ZFace tracker to detect faces in video sequences. For classification, a set of classifiers is trained, including NB, kNN, SVM, and RF.

Zhi et al. (73) presented a method for evaluating neonatal pain using dynamic facial representations. For classification, the authors used dynamic facial texture data and geometric features taken from video sequences. Both feature-level and decision-level fusion techniques were employed by the authors. For each type of facial activity classification, the authors have used SVM and have shown results while combining multiple facial activities using a decision fusion scheme (majority voting).

Zamzmi et al. (74) have evaluated N-CNN, a new CNN architecture for assessing pain in neonates proposed in Zamzmi et al. (81). The authors compared the performance of ResNet50 and VGG16 using facial images. To detect the face, the authors have used the ZFace tracker and geometrical augmentations to increase the size of the data samples. Experiments reveal that the proposed architecture is comparable to the other two deep architectures in performance. In another DL-based study conducted, Salekin et al. (75) presented a method for combining information from the neonates' facial expressions and body movements. The authors additionally use LSTM in the proposed multi-channel network to model temporal information. The proposed approach achieved an accuracy of 92.48% and AUROC of 0.90 on video-level classification.

Infants' face muscles are not well developed, according to clinical investigations, and hence their ability to sustain facial actions is limited. Crying sounds are the most common way for infants to express their pain. Zamzmi et al. (71) have presented an automated multimodal approach for assessing neonatal pain that includes crying sounds. Facial expressions, body motion, and vital signs were also added as input features by the authors. Face features were detected, crying sounds were extracted using Yang's speech recognition approach, body motion features were estimated, and the state of arousal was evaluated using facial expression and body motion. The average accuracy of using crying sounds as pain indicators was 88%, and on combining multiple indicators such as facial expression, body motion, and vital signs, the accuracy was increased to 96.6%. The results of the study are promising and could help to enhance the process of measuring neonatal pain.

The authors have presented a comprehensive automatic system that uses the same set of features in another work (76). To generate the pain score, the authors used four fusion schemes: feature-level, decision-level, score-level, and NIPS-based scoring method. The authors have performed experiments using both individual pain indicators and their combination. The highest reported accuracy for the multimodal system was 95.56%.

Salekin et al. (77) did a similar study in which the authors evaluated the N-CNN for measuring pain using crying sounds. The audio signals were converted into spectrogram images by the authors. The authors also compared the performance of the VGG16 and ResNet50 deep architectures. The proposed architecture attained an accuracy of 96.77% and an AUC of 0.94 in experiments. Also, the authors compared their approach with handcrafted features where the results demonstrate that the proposed method outperformed them and achieved an accuracy of 91.20%. The feasibility of employing crying sounds in pain assessment is also demonstrated in this study. Ashwini et al. (79) have reported a strategy in which the authors identified infant crying sounds as hunger, pain, or sleepy using an ML-based classifier. Deep features were extracted and fed to the SVM with different kernels for the classification. Experimental results demonstrate that SVM-RBF achieved the highest accuracy of 88.89% among other variants of kernels in SVM. The authors concluded that using deep features with machine learning classifiers yields good results even with little data samples.

Salekin et al. (78) presented a bilinear CNN with LSTM to assess postoperative pain in newborns. In addition to modeling temporal pain, the authors looked at facial features. The authors used bilinear CNN to extract features relating to distinct pain intensities from both acute and postoperative pain data. The authors achieved an MSE of 3.999 and an MAE of 1.5565. The analysis of the results also demonstrates the feasibility of this framework in assessing newborn postoperative pain. Salekin et al. (80) did a similar study in which the authors presented a multi-modal spatio-temporal technique for assessing postoperative pain in neonates using visual and verbal indicators. The authors have used VGG-NET for feature extraction, followed by classification using Bilinear CNN. Extensive experiments are performed by authors using a single modality and multiple modalities. The authors concluded that the multimodal approach is more reliable and feasible to deploy in a real-world environment.

### 3.2. Predicting Sepsis in Neonates

Infections affect people of all ages, but they are particularly risky in infants because their immune systems are still developing and, thus, more prone to diseases. Although certain defensive antibodies transfer from the mother to the baby *via* the placenta (the organ that feeds the fetus), the amounts of antibodies in the fetus's bloodstream may not be sufficient to combat an infection. Infections can be acquired by fetuses and neonates either during pregnancy and birth or following birth. A systemic inflammatory response to an infection is defined as sepsis. It is related to the high mortality and morbidity rate. Both adults and children get affected. Early detection can help in reducing mortality and morbidity. For the early detection of sepsis in newborns, the following studies are being done. Table 9 summarizes the approaches.

**Table 9:** Summary of approaches used to predict neonatal sepsis.

**References**	**Summary**	**Key Contribution**	**Dataset used**
Mani et al. (82)	Proposed a machine learning approach to predict late-onset neonatal sepsis using electronic medical records	The proposed approach can prove helpful in identifying truly infected neonates and can act as an early warning system. Detected the top three sepsis predictive variables as packed cell volume, chorioamnionitis and respiratory rate.	Collected 299 samples of neonates for late-onset sepsis from the Monroe Carell Jr. Children's Hospital
Le et at. (83)	Proposed an ML-based sepsis prediction system for neonates using machine learning	This system can help in continuous monitoring of EHR data and hence the probability of developing the sepsis in neonates Use of vital signs further improves the model performance	Used de-identified chart data from UCSF where the age of the patients ranges between 2 and 17 years
Masino et al. (84)	Evaluated various ML-based algorithms for the prediction of neonatal sepsis	The authors have studied the feasibility of using machine learning to develop early neonatal sepsis prediction models. Logistic regression can generalize well with other EHR datasets with the same input features and is resilient to overfitting.	Collected data from patients who were hospitalized for at least 48 hrs in the NICU (in CHOP) and also have received at least one sepsis evaluation before 12 months of age

Using electronic medical records, Mani et al. (82) presented a non-invasive strategy for late-onset newborn sepsis. The authors used a variety of classifiers, such as NB, SVM, kNN, and others. To deal with missing values, the authors utilized a single imputation strategy, in which a random number is generated for continuous variables based on the mean and standard deviation of the observed values. Imputation for discrete values is done by selecting random values from a set of observed discrete values weighted by their proportion. The results demonstrate that the proposed method outperforms the decision of clinicians in terms of sensitivity and specificity. The authors of Le et al. (83) conducted a similar study in which they employed ensembles of decision trees to predict sepsis in neonates. The results demonstrated that at the time of onset, the algorithm achieved an AUROC of 0.916 for classification between severe sepsis and control pediatric patients and an AUROC of 0.718 at 4h before onset. Their technique outperforms existing sepsis scoring systems, such as pediatric organ failure and inflammatory response scoring systems, according to experimental results. Masino et al. (84) have evaluated the machine learning algorithms for the prediction of neonatal sepsis using electronic health records. The authors have employed 8 machine learning models for classification, out of which 6 models achieved a mean AUROC between 0.80-0.82. Early prediction can help in reducing the need to give antibiotics if found negative for sepsis.

### 3.3. Predicting Jaundice in Neonates

Jaundice is a common health condition that affects people all over the world. Both adults and children are affected. It results in yellowing of the skin and is generally visible in the eyes and skin. Jaundice in infants is prevalent, especially in babies born before 38 weeks of pregnancy (preterm babies) and some breastfed babies. When a baby's liver isn't developed enough to get rid of bilirubin in the bloodstream, it causes jaundice. A timely diagnosis can lead to a more accurate diagnosis and the avoidance of negative consequences such as neurological disorders. Existing bilirubin level estimation approaches are invasive, requiring blood to be extracted from the patient's body and a diagnostic test to be done. The issue arises when these levels must be monitored on a regular basis.

To help in this direction, the research community is attempting to develop non-invasive, cost-effective solutions. These approaches can assist resource-constrained communities in reducing the requirement for equipment that is scarce and limited in such settings. Table 10 summarizes the approaches, including the key contribution and the dataset being used. Taylor et al. (85) presented a smartphone-based application for estimating bilirubin levels in newborns. The authors used images taken using smartphones and a calibration card to ensure color consistency. The results demonstrate that the TSB levels and the predicted value have a strong correlation of 0.91. The sensitivity and the specificity of the app were 84.6 and 75.1%, respectively. The results demonstrate that this app can aid in detecting children that need medical attention. Leung et al. (86) proposed to use sclera images to assess neonatal jaundice. The authors used multiple linear regression to find the correlation between pixel values (RGB) of the sclera and the Total Serum Bilirubin (TSB) of the neonates. A comparison of estimated and measured TSB levels is also performed. The results show that r = 0.75 and that their method has a sensitivity of 1.00.

**Table 10:** Summary of approaches used to predict neonatal jaundice.

**References**	**Summary**	**Key Contribution**	**Dataset used**
Taylor et al. (85)	A smartphone-based app called BiliCam to estimate the bilirubin levels in neonates is proposed	A technology is proposed based on images to estimate the TSB values in neonates. Can act as a screening device to help identify the neonates that require blood draw. Accurately identifies neonates with high TSB levels	Collected 580 samples of newborns (<7 days old) at 7 sites across United States
Leung et al. (86)	The authors proposed a neonatal jaundice screening method using sclera images	The authors proposed a smartphone-based approach based on two color spaces (RGB and CIE XYZ) that can quantify the yellow color of the sclera. A new grading scale, JECI, is introduced that helps to quantify yellow color and is also device-independent. JECI can be helpful in the screening of jaundice in adults as well.	Collected 87 images of neonates whose age was between 1 day and 28 days (in UCL Hospital)
Aune et al. (87)	A color analysis based solution is proposed to estimate bilirubin levels in neonates using smartphone-captured images	This approach can detect severe jaundice with high sensitivity and also shows that a calibration card can minimize the effect of varying illumination. Limitation: Their dataset mainly contains Caucasian neonates; hence the learnt model may not work well with non-caucasian infants and there was no consensus between sites for data collection.	Collected images of 302 neonates having up to 15 days of age from 2 hospitals (in Norway)
Outlaw et al. (88)	A smartphone-based solution for the screening of jaundice in neonates using sclera and conjunctiva images is proposed	The authors employed ambient-subtracted scleral chromaticity to describe the color of modality to quantify neonatal jaundice, which eliminates the need for color calibration. The results show that linear models based on scleral chromaticity are capable of accurately estimating TSB.	Collected data of 51 neonates (in UCL Hospital) whose gestational age ranges from 35 weeks and 6 days to 1 week and 1 day
Althanian et al. (89)	Proposed a multi-modal approach to detect jaundice in neonates	A predictive model based on a set of modalities such as skin, eye and their combination is proposed to diagnose jaundice in neonates Concluded from results that skin and eye features work best with deep models and traditional machine learning, respectively. The best set of features may not be the best for all classifiers	Collected dataset of 100 neonates (in KKU Hospital in Riyadh) whose average gestational age was 38 weeks and the average age was 1 day

Aune et al. (87) performed a similar study in which the authors used color analysis of skin images to assess bilirubin levels. To minimize the effect of ambient illumination, the authors used images with and without a flash of the smartphone. The reported results demonstrate that the sensitivity and specificity of the proposed approach were 100 and 69%, respectively. Furthermore, the authors reported a 0.84 correlation between the estimated value (smartphone) and the TSB and also a correlation of 0.81 between image estimates and TcB, demonstrating the approach's practicality. Outlaw et al. (88) have presented a smartphone-based method for estimating neonatal bilirubin levels. To achieve color consistency, the authors adopted the ambient subtraction method, which eliminated the requirement for any external attachment like a calibration card. The results demonstrate that the proposed method can achieve a sensitivity and specificity of 100 and 61% for infants with TSB above 250μmol/L respectively, and a sensitivity and specificity of 100 and 54% for infants with TSB levels below 250μmol/L.

To propose a multi-modal system and improve the accuracy of the approach, Althanian et al. (89) have proposed to use eye, skin and their combination as the input. The authors have used various image processing techniques like color balancing etc, in this work. The authors have evaluated machine learning algorithms such as MLP, SVM, DT, and RF in addition to deep learning. The results demonstrate the transfer learning approach achieved accuracy and AUC of (86.83%, 81.05%), (79.03%, 69.67%), (79.95%, 71.25%) for skin, eye, and their fused features, respectively, showing that skin features work well with deep models.

### 3.4. Machine Learning in Tracking Malnutrition

A well-balanced diet is an important component of living a healthy lifestyle. Malnutrition is a global health issue that manifests itself in a variety of ways, including undernutrition, overweight, obesity, and a lack of vitamins and minerals. Malnutrition is directly or indirectly associated to about half of infant mortality in underdeveloped nations, according to Pelletier and Frongillo (90). Malnourished children are more susceptible to infections, weight loss, obesity, and other ailments. Malnutrition can be caused by a variety of causes, including demographic, socioeconomic, health, and physical factors. As a result, the features that constitute malnutrition must be identified. Researchers are concentrating their efforts on creating techniques for predicting malnutrition. In the literature, there are several approaches that employ statistical tools to explain the factors that contribute to malnutrition. The most common methods for predicting the probability of malnutrition in children are linear regression and logistic regression.

Khare et al. (91) studied the relationship between childhood malnutrition and socioeconomic factors. On the identified explanatory features, the authors utilized a logistic regression model. Their research found that machine learning approaches are useful in identifying the most important variables that contribute to malnutrition prediction. Various research studies from different regions also show that AI technologies may be used to predict malnutrition (92–96).

Lingren et al. (97) have provided a method for identifying obese children. On the EHR records, the authors applied rule-based and machine learning techniques. The chi-square approach is used to select features. The approach was also tested utilizing NB. The findings show that their technique has the potential to be employed in clinical trials.

## 4. Challenges and Path Forward

### 4.1. Economic and Social Barriers

Although the maternal mortality rate (MMR) is one of the most critical indicators of a nation's maternal well being, MMR accounts for only a tiny portion of the strain on maternal illnesses, which refers to the medical conditions women face during gestation and the postpartum period. For every female dying from pregnancy-related reasons, another 20 or 30 suffer from underlying medical illnesses, frequently with long-term consequences that impair their ability to function normally (98, 99). These consequences can impact the individual's physical, cognitive, and reproductive health and their ability to perform in specific domains (e.g., intellect, mobility, and socialization), self-image, and cultural-financial standing (99, 100). Maternal mortality and other related issues are expected to be highest in low- and middle-income nations, particularly amongst some of the poorest women families (101). The actual cost of maternal deaths, on the other hand, is unknown. Current estimations and computations are not founded on well-maintained records, techniques etc. Such strategies are ineffective and have low validity in informing initiatives to solve maternal diseases. The lack of a clear definition and standardized identification procedures is one of the main reasons for the challenges in effectively quantifying maternal malaise. Unreliable vital statistics aggravate this issue due to insufficient healthcare information systems (HIS).

Premature birth, low birth weight infants, fetal growth restriction, antenatal complications (e.g., anemia, eclampsia) and other factors during delivery (e.g., extended labor, umbilical cord prolapse) contribute to the development of neonatal health risks such as cerebral palsy, learning disabilities, and other abnormalities. Underskilled caregivers and health workers, such as relatives and midwives, may not be able to recognize and manage intrapartum-related problems in an infant in low resource settings. This reveals a gap between both the commencement and acknowledgment of the problem and seeking appropriate treatment. In the case of intrapartum difficulties, prompt recognition and reaction are critical since even a minor delay in seeking and obtaining adequate healthcare can result in significant impairment to the infant, potentially resulting in life-threatening situations (102).

### 4.2. Technological Barriers

Even though these technologies are quickly advancing, their clinical use is still in its early stages. A comprehensive validation study is required before clinical clearance for employing AI-based models to assess the applicability of the proposed approach in practical clinical scenarios. Before a model can be implemented in the healthcare area, it is necessary to develop standard validated tools for its use in actual healthcare settings. A few of the technological barriers to wider applicability and acceptance of AI in the clinical scenario are as follows:

- Non-Availability of large scale databases - Whether it is extracting healthcare data, conducting survey-based studies, or studying omics, proteomics, and other data, a majority of existing efforts are laborious and time-consuming. New cutting-edge AI/ML-based technologies are emerging as solutions to the overburdened healthcare paradigm. Their widespread adoption lies in the fact that these technologies are deployable in maternal and neonatal health without requiring too much clinical information about the subjects. However, these technologies need to be trained on information from a large volume of patients to achieve this. This kind of big data collection poses unprecedented issues for storing, handling, transferring, and securing these datasets and ensuring patient privacy.
- Evaluation and validation on a uniformly sampled database which is a true representative of the population - There is also rising concern about the possibility of bias in the AI-based predictions, all of which are contributing to this problem.
- Trustworthiness and explainability of the models - At the moment, the lack of explainability in machine learning prevents the widespread adoption of artificial intelligence. Explainability is especially required in healthcare as here; the medical diagnosis model is accountable for human life. Suppose artificial intelligence (AI) is unable to explain itself in the domain of healthcare. In that case, the risk of making the wrong judgement may outweigh the benefits of precision, rapidity, and judgement efficacy. As a result, the breadth and utility of the system would be significantly restricted. Thus, it is critical that these issues are thoroughly investigated (103).
- Concerns about privacy - AI has often raised concerns in the minds of users regarding their stored data privacy (due to AI requiring big-data to train on) as well as the privacy of the predictions made by AI. For example, the predictive power of AI in healthcare can aid in giving diagnosis to a disease the patient has never disclosed to anyone or didn't even know himself. Here, it's important to store and use this data ethically with proper consent (104).
- Acceptance of AI models and samples integration in daily procedures - The larger community must embrace new tools and drive training, patient participation, and rigorous standard development to enable more methodical collaboration across hospitals. Organizations must contribute to the purposeful and thoughtful creation of medical environment models. Advancements in the machine learning sector will surely assist in shaping future healthcare discoveries if we can achieve this.

### 4.3. Open Problems for Research

As discussed above, limited access to proper healthcare infrastructure and professionals, along with other societal and economic barriers, put restrictions on providing holistic care to pregnant women and their children in resource-constrained settings. Digital chatbots and support groups can aid in maternal and neonatal health monitoring as well as management (105, 106). One key challenge here lies in handling the cultural diversity of the users pertaining to the language they are comfortable conversing in. Thus, chatbots can be developed to engage the users in follow up questions about their health in their desired language. These conversational healthcare technologies come under the domain of Natural Language Processing (NLP) in AI and help in dispersing basic health information amongst the users.

Explainable Artificial Intelligence (XAI) or Dependable AI (DAI) can be used to give insights into the decision-making process of the AI models. Explainability raises the level of confidence that medical practitioners and AI researchers have in an AI system, leading to more broad adoption of AI in the healthcare field over time. For example, if they explain why someone has been classified as ill or otherwise, we can look inside the programmed thought process of the model. It would change the perception about these models as a “black box” and help in their scalable employment (103). Eventually, XAI can be merged with smart healthcare systems that incorporate the Internet of Things, cloud computing, and artificial intelligence, and which will be particularly applied in the fields of maternal and neonatal health. These intelligent healthcare systems can therefore be utilized for a variety of purposes, including the diagnosis of diseases and the selection of suitable treatment plans (107).

To supplement the lack of data in some crucial small sample size healthcare problems where the large dataset is not available, Few-shot learning or Zero-shot learning can be used to train the AI models. Newer and more efficient few-shot learning frameworks for healthcare are needed to be developed to utilize domain information to reach medical decisions/ predictions. These technologies would be an efficient assistive tool for doctors to handle rare medical conditions and problems that require years of experience (108).

## 5. Summary

The MDGs stated that one of the primary objectives of world leaders was to improve maternal health and reduce child mortality. In 2015, as part of the 17 Sustainable Development Goals, this objective was expanded to include the reduction of maternal and newborn mortality due to problems during pregnancy and childbirth. Improving maternal and newborn health entails bringing speedy diagnosis and treatment to point-of-care settings in developing nations with limited resources. However, there are currently just a few diagnostic tools and techniques available in point-of-care settings. This is by far the most common cause of maternal and newborn death. The purpose of this study is to examine how artificial intelligence and machine learning are being used to improve currently developing technologies, revealing critical gaps in development where novel design could boost access to technology and enable rapid diagnosis at the bedside. Issues pertaining to maternal and neonatal health are often not addressed due to a lack of awareness in the social caregivers to recognize the early signs of the impairment. The lack of skilled, professional healthcare workers also devoids proper demographic coverage, and many such health issues go unaddressed or, even worse, unaccounted for. This gives rise to unreliable data collection and, in turn, leads to delays in action to improve health amenities. With the help of AI/ML algorithms, we can address many of these issues; however, the need for explainability in AI is a major roadblock to its widespread adoption. When implementing AI solutions for public health, the ethical concepts of a sense of morality, autonomy, and justice, as well as human rights such as respect, independence, well being, self-determination, fairness, equality, and privacy, must all be taken into account. Once we are able to jump across these hurdles, not only can we reduce the workload of doctors and healthcare workers, we can accumulate reliable data with wide demographic coverage and can see the girth of the problem. This way, we can more efficiently aid in improving maternal and neonatal health, especially in low income and poor resource settings which are otherwise untouched by proper facilities and remedies. Future work on making AI-based techniques explainable would ensure more confidence of health practitioners in this technology and will hopefully help in the large scale adoption of AI in this sector.

## Author Contributions

MKha and MKhu took the lead in writing the paper. MV, RS, MD, and KS conceptualized the idea and reviewed the paper. All authors contributed to the conception of the idea.

## Funding

This research was supported by a grant from iHub-Drishti, IIT Jodhpur.

## Conflict of Interest

The authors declare that the research was conducted in the absence of any commercial or financial relationships that could be construed as a potential conflict of interest.

## Publisher's Note

All claims expressed in this article are solely those of the authors and do not necessarily represent those of their affiliated organizations, or those of the publisher, the editors and the reviewers. Any product that may be evaluated in this article, or claim that may be made by its manufacturer, is not guaranteed or endorsed by the publisher.
